# Spontaneous Bowel Degloving and Evisceration Through Uterine Perforation Due to Unsafe Abortion: A Case Report

**DOI:** 10.7759/cureus.90678

**Published:** 2025-08-21

**Authors:** Aneesh Kumar, Satyendra K Tiwary, Puneet Kumar, Ajay Khanna

**Affiliations:** 1 Department of General Surgery, Institute of Medical Sciences, Banaras Hindu University, Varanasi, IND; 2 Department of Surgery, Institute of Medical Sciences, Banaras Hindu University, Varanasi, IND

**Keywords:** abortion, bowel degloving, bowel evisceration, suction and evacuation, uterine perforation

## Abstract

Induced abortion is a common surgical procedure worldwide, regardless of whether it is performed for therapeutic or elective purposes. It contributes significantly to maternal mortality and morbidity. Uterine perforation is one of the major consequences that can occur while performing the procedure under unsafe conditions by unqualified personnel. It becomes a surgical emergency when bowel prolapses through the vaginal introitus. We present a case of a 35-year-old lady with bowel without its mesentery prolapsing through her vagina following a procedure of suction and evacuation for incomplete abortion. She underwent an exploratory laparotomy with segmental resection of the degloved large bowel from the cecum to the rectosigmoid junction. The distal healthy stump, located at the rectosigmoid junction, was closed primarily, and the ileum was divided at the ileocecal region (ICR) and brought out as a permanent end ileostomy, and primary closure of the uterine perforation was done. It is advised to manage it using a multidisciplinary approach. To raise awareness of these deadly complications and recognize high-risk and challenging cases, training programs must be organized.

## Introduction

Any intrauterine procedure carries the risk of uterine perforation, which might harm surrounding blood vessels or the viscera (bowel, bladder) [1]. The incidence of uterine perforation is low, occurring in less than 0.3% of procedural abortions performed in the first and second trimesters [2]. Insufficient cervical dilatation prior to surgery and the inexperience of the surgeon are linked to a higher risk of perforation during pregnancy termination [3]. The risk of uterine perforation is increased by conditions including cervical stenosis, strong anteflexion or retroflexion that hinder access to the endometrial cavity, or pregnancy, lactation, menopause, or prior uterine disruption that compromises the myometrial wall's strength. Since most illegal abortions are carried out in remote locations, frequently in unhygienic settings, by people with a lack of anatomical training, and with non-sterile equipment, the death and morbidity rates are higher. Bowel degloving and evisceration following surgical abortion due to uterine wall perforation is a rare but dangerous consequence. It is a true emergency because of the numerous potentially lethal surgical outcomes [4]. This case study follows the Surgical CAse REport (SCARE) checklist [5].

## Case presentation

A 35-year-old female with an obstetrical score of G4P2L2A1 was referred from a rural private hospital to our tertiary care center with transvaginal evisceration of bowel. She was 14 weeks pregnant, having a history of per-vaginal bleeding for one day, following which she underwent vacuum-assisted evacuation of an incomplete abortion. She had no known chronic illnesses, trauma, or history of previous surgery.

**Figure 1:**
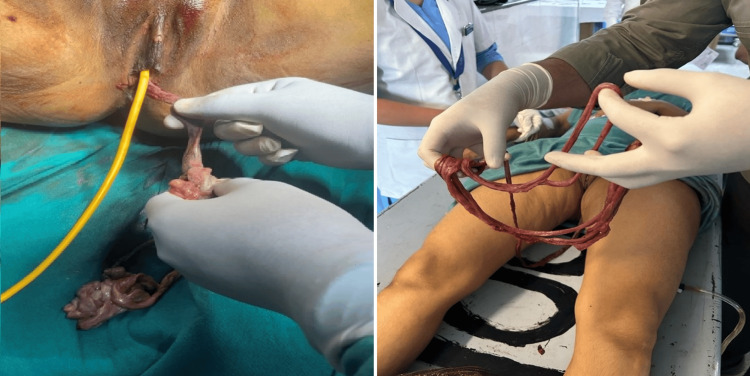
Prolapse of bowel without its mesentery through the vagina, giving a tether-like appearance.

On clinical examination, she had a pulse rate of 104 beats per minute, blood pressure measured 116/70 mmHg, respiratory rate was 22 breaths per minute, and her oxygen saturation was 100% on room air. On abdominal examination, there was marked tenderness in the lower abdomen. On pelvic examination, approximately 180-200 cm of bowel without its mesentery, resembling a tether, was prolapsing through the vagina (Figure 1). Digital rectal examination was unremarkable. Laboratory parameters revealed the following: hemoglobin - 8.9 g/dL, total leukocyte count - 11,000/µL, platelet count - 176,000/µL, prothrombin time/international normalized ratio (INR) - 13.7 seconds/1.01, and procalcitonin (PCT) - 1.5 ng/mL. Liver function tests and kidney function tests were within normal limits.

**Figure 2:**
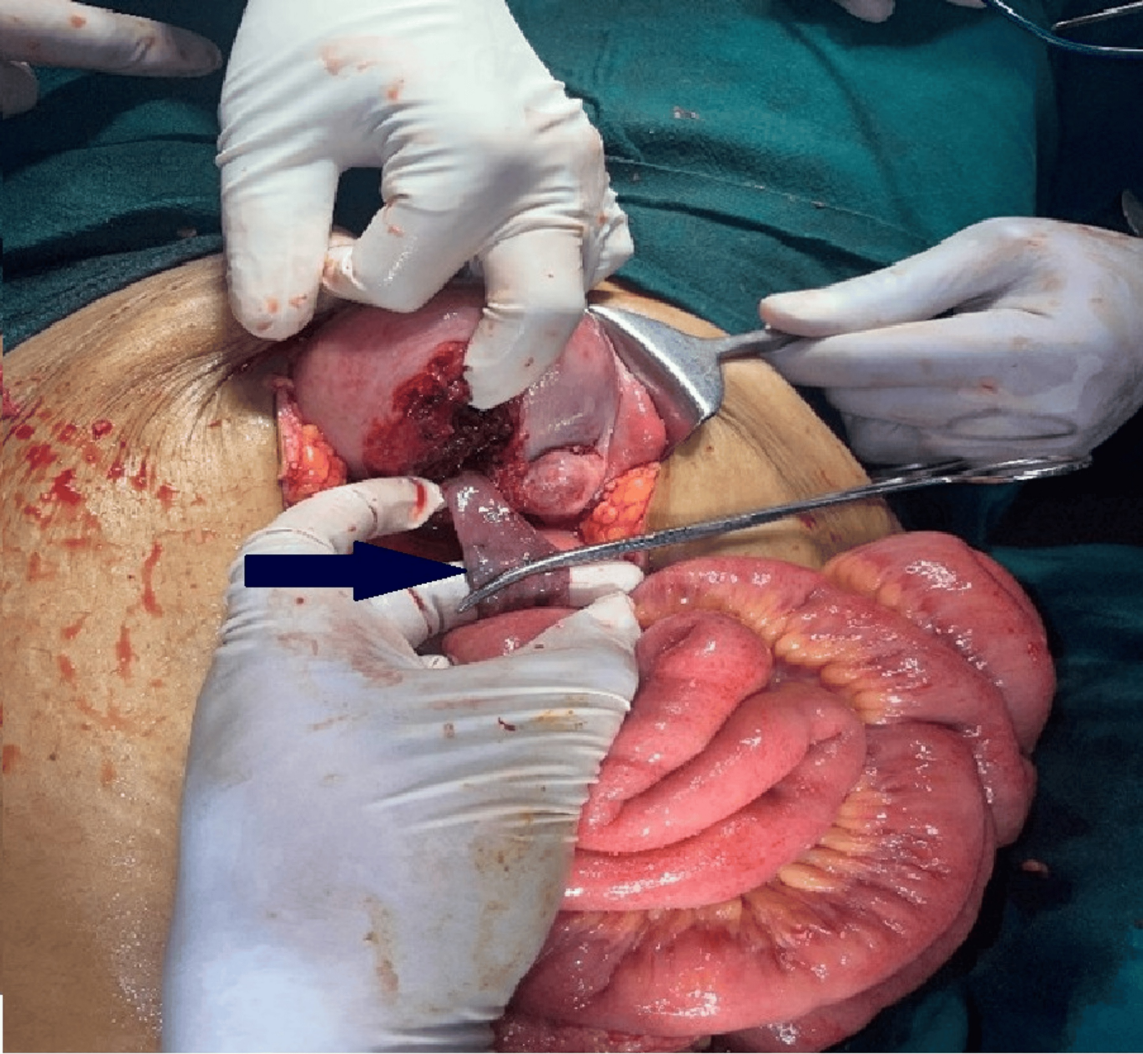
Posterior uterine wall perforation with bowel eviscerating through the defect (blue arrow).

After adequate optimization and fluid resuscitation, she was administered two broad-spectrum intravenous antibiotics. The exposed bowel was covered with sterile, saline-moistened, warm pads, and she was immediately transferred to the operating theater. An emergency exploratory laparotomy was performed via a midline abdominal incision. Intraoperatively, 700 mL of sanguinous content was aspirated from the peritoneal cavity. There was a perforation of approximately 5-6 cm on the posterior wall of the uterus (Figure 2), approximately 6 ft of degloved large bowel (predominantly mucosa) was protruding through it.

**Figure 3:**
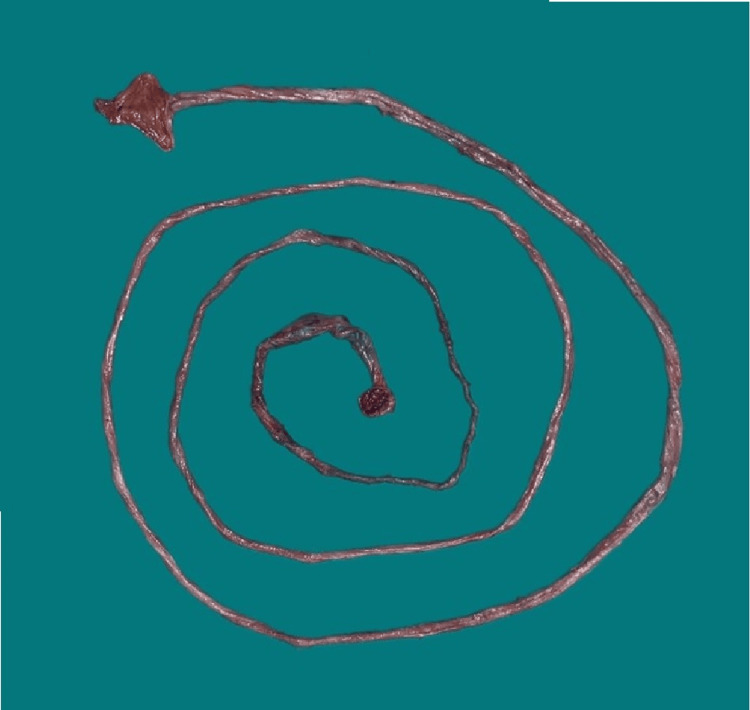
Resected portion of degloved large bowel.

**Figure 4:**
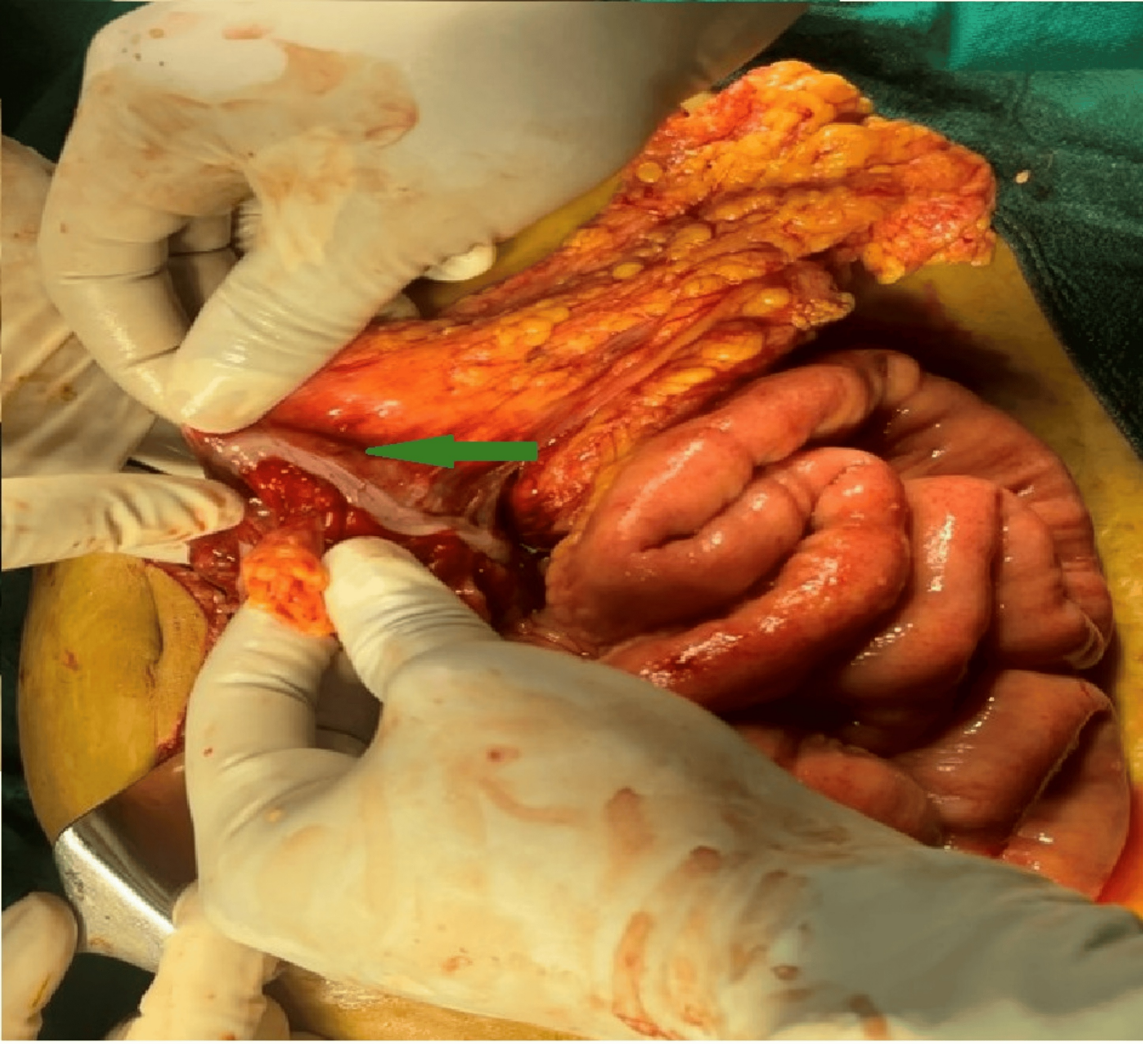
Healthy seromuscular layer of large bowel, without mucosa (green arrow).

**Figure 5:**
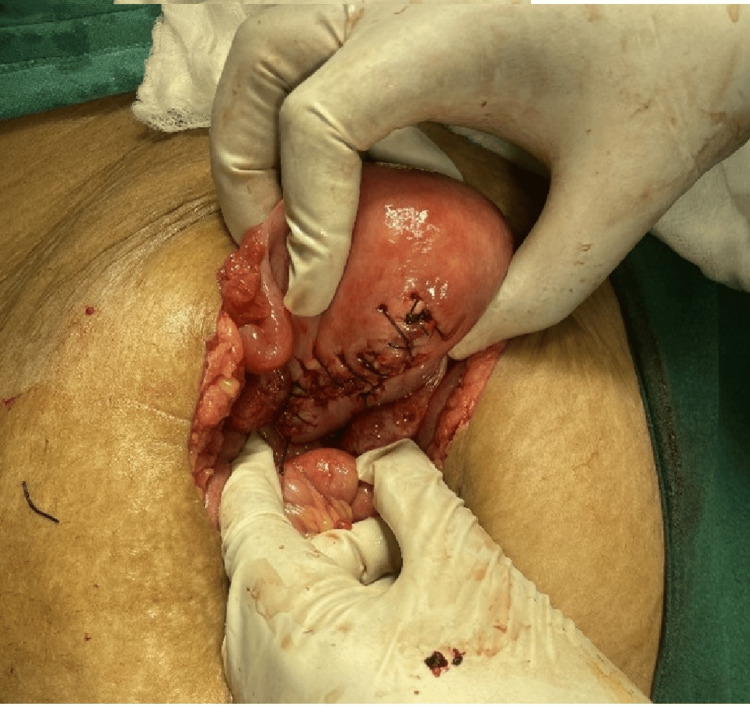
Primary repair of the uterine perforation using interrupted sutures.

The prolapsed segment of bowel was reduced into the peritoneal cavity manually, and segmental resection of the degloved large bowel from the cecum to the rectosigmoid junction was done (Figure 3). The serosa of the large bowel was left in place because it was attached to its mesentery, which appears viable (Figure 4). The distal healthy stump, located at the rectosigmoid junction, was closed primarily and fixed (tucked) to the parietal wall. The ileum was divided at the ileocecal region (ICR) and brought out as a permanent end ileostomy in the right iliac fossa. The stump at the ICR was also closed primarily. The uterine perforation was closed primarily with an interrupted absorbable Vicryl 1.0 suture (Figure 5).

The patient had an uneventful postoperative recovery and was discharged on postoperative day 9 with recommendations.

## Discussion

In the literature, rarely few cases of intestinal evisceration following vacuum-assisted abortion or delivery have been documented. One of the more common and dangerous consequences of intrauterine procedures is uterine perforation. Following a gynecological operation, it is defined as the disintegration of the uterine wall's entire thickness [6]. Depending on the type of intervention, the prevalence rate associated with uterine perforation varies, like 1.6% after a hysteroscopy [7] (typically for therapeutic purposes), 0.5% after an induced or spontaneous pregnancy termination [8], and about 5% during the removal of retained products of conception performed to manage postpartum hemorrhage [5]. The anterior uterine wall accounts for 40% of uterine perforations, cervical canal (36%), and fundus in 13% [9].

Risk factors include uterine abnormalities (malposition or anatomical distortion), a uterine scar, inadequate cervix preparation before an intrauterine diagnostic or therapeutic operation, and inflammation [6]. Bowel involvement can be categorized into four clinicopathological categories based on severity: obstruction, strangulation, mesenteric stripping, and bowel degloving [10].

The uterine wall or pelvic viscera (intestines, omentum, or ovaries) can be immediately visualized through the intraoperative breach to aid in an early diagnosis [7]. It may be detected when the instrument extends beyond the fundal length during the procedure, or when there is injury-related loss of resistance or visceral visualization through the breach. A uterine perforation may be treated conservatively or surgically, depending on the abdominal visceral injury and the clinical signs (bleeding) [6]. It is preferable to resuscitate the patient as soon as possible.

Conservative therapy is indicated in cases where the patient is asymptomatic and if a blunt instrument, such as a dilator, suction-free curette, or hysteroscope, was used without an electrosurgical energy source. A urinary catheter is inserted as part of the conservative treatment, along with antibiotics and hospital monitoring for intestinal obstruction, peritonitis, or bleeding indicators such as abdominal pain, distention, and hemoglobin and hematocrit levels.

Surgical exploration is indicated when there is a risk of vascular or visceral damage, when suction or sharp objects are employed, when there is a perforation following a pregnancy termination, or when there is significant and ongoing uterine hemorrhage [9]. Bowel evisceration in the perineum is a definite indication for surgery. Because laparoscopy has a lower risk of perioperative morbidity than laparotomy, it is the preferred method for surgical exploration if the patient is stable. Laparotomy may be beneficial for patients who are not hemodynamically stable [6,9].

## Conclusions

Unsafe abortions continue to be a serious public health issue, particularly in low-income nations. It increases the risk of complications that could result in maternal morbidity or death, such as septic shock, peritonitis, uterine perforation with bowel evisceration into the perineum, and multi-organ dysfunction. It is recommended to handle it with a multidisciplinary strategy. It emphasizes the need of having qualified medical personnel to perform these interventional treatments. Training programs must be organized to increase awareness of these fatal consequences and identify high-risk and challenging cases.
